# Editorial: Animal welfare science: Rising to the challenges of a changing world

**DOI:** 10.3389/fvets.2023.1151773

**Published:** 2023-02-15

**Authors:** Keelin Katherine Mary O'Driscoll, Fidelma Butler, Gareth Arnott

**Affiliations:** ^1^Pig Development Department, Moorepark Animal and Grassland Research Centre, Teagasc, Fermoy, Ireland; ^2^School of Biological, Earth and Environmental Sciences, University College Cork, Cork, Ireland; ^3^School of Biological Sciences, Queen's University Belfast, Belfast, United Kingdom

**Keywords:** animal welfare, technology, PLF, sow, one welfare

We are living in a time of significant global challenges and changes. Improving animal welfare can help in tackling many of these, so the science of animal welfare has a large part to play in helping attain a sustainable way of living in the future. There is also growing awareness and concern amongst many people around the world regarding the treatment and welfare of animals, and our responsibilities toward them. This has led to a growing shift in focus away from simply ensuring that managed animals do not have poor welfare, and toward providing them with a good quality of life. This Research Topic contains a selection of papers submitted to the 8th International Conference on the Assessment of Animal Welfare at Farm and Group Level (WAFL) which address these challenges. The collection includes a review of the challenges posted by precision livestock farming for sustainable livestock production (Tuyttens et al.), as well as two other papers demonstrating how technologies such as cameras can also present opportunities to reduce workload (Cooke et al.), and handling stress on animals (Qin et al.). It also includes a review discussing the impact of housing on sow welfare during the transition from lactation to the subsequent pregnancy, identifying a dearth of scientific knowledge in this area (Chou and Parsons). Finally, we also include a paper demonstrating how management and the environment can impact on animal resilience (Luo et al.).

## Author contributions

KO'D wrote the first draft and the other authors edited and approved the manuscript.

